# Behavioral change in waste separation at source in Chile: an application of the theory of planned behavior

**DOI:** 10.3389/fpsyg.2026.1846103

**Published:** 2026-07-06

**Authors:** Jean Pierre Doussoulin, Franz Carrillo-Higueras, Fernanda Mera, Alejandra Navarro, Linda Calderon-Vera

**Affiliations:** 1 ERUDITE (EA 437), Equipe de Recherche sur l'Utilisation des Données Individuelles en lien avec la Théorie Economique, Université Gustave Eiffel, Champs-sur-Marne, France; 2 Facultad de Ciencias Económicas y Administrativas, Universidad Austral de Chile, Valdivia, Chile; 3 Escuela de Administración de Servicios, Universidad de los Andes, Santiago, Chile; 4 Faculty of Education and Social Sciences, Observatos Research Group, Tecnológico de Antioquia, Medellín, Colombia

**Keywords:** governance and Chile, home waste separation, municipal waste management, structural equation modeling (SEM), theory of planned behavior, circular economy

## Abstract

Understanding how individuals engage in waste separation is a central challenge in sustainability and waste management, particularly in relation to their perceptions of public institutions. While governments and municipalities have implemented multiple initiatives to promote pro-environmental behavior, the effectiveness of these efforts is closely related to the behavioral factors associated with household decision-making. This study examines the key factors associated with waste separation intentions in several cities in southern Chile using an extended Theory of Planned Behavior (TPB) framework.

The survey instrument incorporates additional constructs, including information availability, perceived role of municipalities and willingness to pay. Using structural equation modeling, the results indicate that subjective norms and perceived behavioral control are strongly associated with behavioral intention, while attitudes are not significantly related to intention. Information provided by municipalities is indirectly associated with intention through its relationships with attitudes and perceived behavioral control.

The findings highlight the relevance of social influence, perceived feasibility, and access to information as factors associated with pro-environmental intentions. However, given the cross-sectional design of the study, these results should be interpreted as associations rather than causal relationships or observed behavioral change. These insights contribute to the extension of TPB in the context of environmental behavior and provide indicative implications for the design of waste management policies.

## 1 Introduction

Solid waste management is a global challenge that affects individuals, communities, and governments (Doussoulin, 2021). Daily productivity, cleanliness, and public health can all be negatively affected by inadequate or inefficient waste management practices (Alao et al., 2025). The complexity of environmental problems related to urban metabolism, such as waste management, requires a transdisciplinary approach (Keskin and Mengüç, 2018; Hofmann et al., 2025; Moosavi et al., 2025). This perspective should incorporate not only the economic and technical aspects typically addressed in environmental sciences, but also the perspectives of users at both the individual and municipal levels, including intentions, attitudes, and perceived behavioral control that influence environmental behaviors (Ma et al., 2018). Understanding which of these components is the most influential at the territorial or municipal level allows policymakers to better target public policies toward specific socio-economic groups.

Several studies that apply the Theory of Planned Behavior (TPB) to analyze environmental behavior, such as the one by Wang et al. (2021), include the term “municipal” in their titles, and since municipalities typically play a key role in urban waste management. We argue that it is important to examine more closely the role of municipal institutions and their influence on individuals' waste-related behaviors.

The article makes several contributions to the fields of environmental psychology and waste management, particularly by bridging the gap between individual psychological factors and institutional governance. Its principal contribution lies in integrating psychological constructs with municipal and informational factors within a Theory of Planned Behavior applied to Chilean cities lacking formal waste separation systems. Several additional contributions are worth highlighting. First, it establishes a direct link between the determinants of individual behavior and municipal public policies, particularly through the roles of information provision and infrastructure. Second, while the traditional TPB focuses on three core constructs, this study extends the framework by incorporating information availability, perceived municipal role, and willingness to pay (WTP) into the structural equation model.

Finally, whereas much of the existing literature focuses either on developed countries with well-established waste separation systems or on large metropolitan areas in developing contexts, this study examines medium-sized cities in southern Chile (specifically Coyhaique, Panguipulli, and Valdivia) where formal municipal waste separation systems are still under development.

Ma et al. (2018) argued that behavioral intention toward waste separation at the source can be considerably predicted by people's positive attitudes toward this practice. Similarly, Shen et al. (2019) demonstrated that when locals observe others in their immediate surroundings waste separation their waste and feel pressured to do the same, they are more likely to engage in this behavior themselves. Perceived behavioral control also influences behavior and is shaped by individuals' perceived abilities and confidence to perform the action (Bandura, 1986).

Stoeva and Alriksson (2017) suggests that public accessibility and information dissemination significantly influence people's intention to separate waste. More recently Hu et al. (2026) mentions that external factors like infrastructure and education support sustained behavioral change. In addition, research on waste separation behavior in Hong Kong (Wan et al., 2014) showed that regulations that can be proposed and enforced by the state play an important role in directly or indirectly creating more waste recyclers. The novelty of the present study, compared with previous research such as Novanda et al. (2026), Concari et al. (2022), Phulwani et al. (2020), Shi et al. (2021), and Si et al. (2019), lies in the explicit incorporation of the municipal role as a variable in the analysis. This perspective links individual behavioral determinants with municipal public policies related to waste separation.

To identify the factors that predict residents' intentions and behaviors regarding waste separation at the source, questionnaire surveys are commonly used in studies conducted within the TPB framework (Oztekin et al., 2017), which was the selected way of collecting information for this research. Given the growing concerns surrounding waste management, further research is needed to develop more effective approaches that prioritize sustainability and the environmental wellbeing of citizens.

## 2 Background

### 2.1 Environmental science and governance toward TPB

Environmental science is an interdisciplinary field that examines the interactions between living organisms, including humans, and their environment. More recently, it has expanded to incorporate emerging challenges related to technology and artificial intelligence (Shuford, 2024). Its central objective is to understand and address environmental problems while promoting sustainability and conservation (Allaby, 2002; Chiras, 2009).

**Figure 1:**
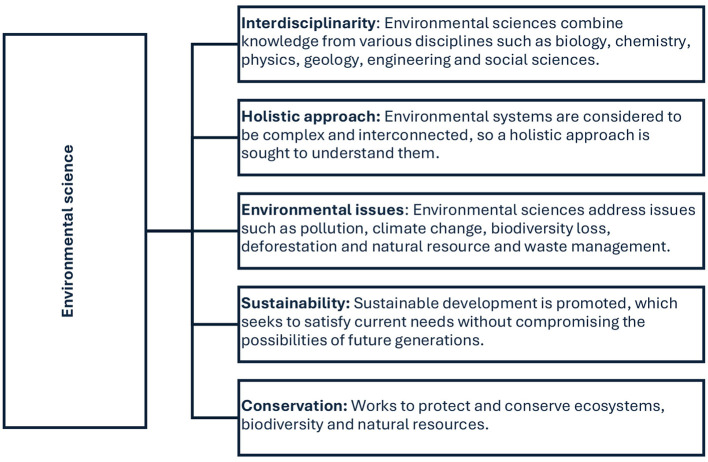
Environmental science key issues. Source: (Allaby, 2002; Chiras, 2009).

Given its holistic nature, environmental science has increasingly adopted a governance-oriented perspective, recognizing that environmental challenges are not only technical but also political and institutional. As noted by O'Riordan (2004), environmental science has become deeply embedded in political processes, particularly at the local level, where sustainability initiatives are implemented and contested. In this context, sustainable development has emerged as a key organizing principle, closely linked to evolving forms of governance (Jordan, 2008). Figure 1 shows key issues in environmental science.

Contemporary governance frameworks emphasize multilevel and decentralized processes that involve actors across national, regional, and local scales. According to Hooghe and Marks (2001), governance extends beyond formal state structures to include complex arrangements of authority distributed both upwards and downwards. At the subnational level, this has led to the consolidation of more pluralistic and participatory governance systems, characterized by horizontal collaboration and multilevel coordination (Delamaza et al., 2015; Menocal and Eade, 2004). These dynamics are particularly relevant in environmental management, where local governments play a critical role in policy implementation and citizen engagement.

Waste governance exemplifies this increasing complexity. Over recent decades, rising waste generation, environmental concerns, and growing social awareness have intensified the need for institutional reform and more effective public policies (Abreu et al., 2024; Yang et al., 2024). Municipalities, in particular, have become central actors in designing and implementing waste management systems, as they operate at the interface between policy and household behavior. Despite these efforts, significant challenges remain, as both institutional constraints and behavioral factors influence the effectiveness of waste management strategies.

One of the key challenges in this domain is promoting waste separation at the source, which is a fundamental component of sustainable and circular waste management systems. Several developed countries—including Germany, Japan, Singapore, Sweden, and the United States—have successfully implemented such practices, demonstrating the importance of coordinated policy frameworks and citizen participation (Knickmeyer, 2020). However, in many developing and middle-income contexts, understanding and influencing household behavior remains a critical barrier (Oduro-Appiah et al., 2022).

In this regard, behavioral theories offer valuable tools for analyzing and predicting pro-environmental actions. Among these, the Theory of Planned Behavior (TPB) (Ajzen, 1991; Ajzen and Fishbein, 1977) has been widely applied to examine the psychological determinants of behavior, particularly attitudes, subjective norms, and perceived behavioral control. TPB is especially useful in this context because it allows for the systematic analysis of the factors shaping individuals' intentions to engage in waste separation. Furthermore, extensions of the model have demonstrated its capacity to incorporate additional variables—such as information, institutional perceptions, and contextual constraints—enhancing its explanatory power in complex real-world settings (Conner and Armitage, 1998).

Given that municipalities represent the level of government closest to citizens, they are particularly well-positioned to influence household behavior through infrastructure provision, communication strategies, and local policies. Accordingly, this study focuses on the municipal level as the primary context for analyzing waste separation behavior. By integrating governance perspectives with behavioral theory, this research seeks to contribute to a more comprehensive understanding of how institutional and psychological factors interact in shaping pro-environmental behavior.

### 2.2 Theory of planned behavior and people's intentions to recycle and classify waste

Solid waste management (hereafter SWM) has become an increasingly prominent academic topic over the past four decades (Medina-Mijangos and Seguí-Amórtegui, 2020). The literature on SWM adopts multiple approaches to identifying the most appropriate ways to address waste-related challenges. Examples include integrated management (Kannan et al., 2024), waste reduction and prevention targets (Shooshtarian et al., 2024), and zero-waste strategies (Linglin, 2024), among others. A growing and complementary perspective conceptualizes SWM as a governance issue that requires collaborative action and cooperation among all relevant stakeholders (Muheirwe et al., 2024), including both those involved in decision-making and management processes and those affected by the problem (Mujtaba et al., 2024). SWM has long been studied from separate disciplinary perspectives, including economics—through the analysis of incentives and subsidies (Fiorillo and Merkaj, 2024), process engineering and optimization (Rizwan et al., 2020), and public administration and governance (Valkama et al., 2022). However, when examined in isolation, these approaches provide only a partial understanding of the problem. Therefore, this article proposes an integrated perspective that combines the study of waste governance with the analysis of the factors that influence citizens' intentions to separate and sort their waste. In doing so, the study considers both national and municipal contexts.

Numerous studies have examined SWM behaviors from different perspectives. For example, White and Hyde (2012) argue that a direct relationship exists between individuals' intentions to recycle and factors such as attitudes, subjective norms, self-identity, and prior behavior. More recent studies have also explored additional determinants of pro-environmental behavior. For instance, Badawi et al. (2024) analyze the role of awareness in predicting local tourists' plastic waste reduction behavior, while Asyari et al. (2024) examine food waste behavioral intentions in relation to Islamic religiosity. Similarly, Sharma et al. (2024) investigate electronic waste disposal behavior among millennials.

The TPB shows that psychological factors (particularly attitudes, subjective norms, and perceived behavioral control) are important predictors of waste separation intentions. The TPB proposes that behaviors are driven by intentions, which represent an individual's level of willingness to perform a behavior or their perceived likelihood of doing so (Fishbein and Ajzen, 2010). These intentions are influenced by three factors: Attitudes, which refers to how individuals assess the potential outcomes and consequences of engaging in a particular behavior (Ajzen, 1991; Fishbein and Ajzen, 2010). Subjective norms, which is the way individuals interpret the views of important people in their lives regarding the performance of this behavior (Eagly and Chaiken, 1993). And finally, perceptions of control, which are defined as the individuals' evaluations of their own abilities and resources in relation to executing the behavior (Ajzen, 1991).

Several studies have found that people's positive attitudes can strongly affect their behavioral intentions toward domestic waste separation (Kumar and Nandini, 2013; Ma et al., 2018). Zhang et al. (2015) observed that attitudes based on relevant information and moral obligation were the most significant predictors of residents' desire to engage in household waste separation, since there is a preference to engage in good actions or avoid detrimental ones (Ajzen, 1991; Ajzen and Fishbein, 1980).

According to Ajzen and Fishbein (1980), subjective norms are perceived limits and pressures from society that impact people's decision to engage in planned action. Subjective norms are led by normative beliefs in terms of the likelihood of key referent persons or groups accepting or disapproving of such behavior (Ajzen, 1991; Ajzen and Fishbein, 1980). Several studies have shown that people are more likely to recycle, separate and classify waste when they observe others doing so and feel pressure from them (Shen et al., 2019; Stoeva and Alriksson, 2017; Vassanadumrongdee and Kittipongvises, 2018).

Perceived behavioral control refers to individuals' perceptions of the ease or difficulty of performing a particular behavior, which may reflect past experiences as well as anticipated barriers (Ajzen, 1991). Within the framework of the TPB, perceived behavioral control is shaped by control beliefs concerning the presence of factors that may facilitate or hinder the performance of the behavior. These beliefs may be influenced by individuals' previous experiences, their perceived capabilities or confidence in performing the activity, as well as external information or social feedback regarding the behavior (Bandura, 1977, 1986). According to Ajzen (1991), individuals tend to exercise greater control over their behavior when they perceive that more resources, opportunities, and capacities are available and fewer obstacles are present. Empirical studies have shown that perceived behavioral control, particularly in relation to convenience, public accessibility, and the availability of information, has a significant influence on individuals' intentions to separate domestic waste (Pakpour et al., 2014; Stoeva and Alriksson, 2017; Ma et al., 2018).

In addition to the main constructs of the TPB, other factors may also influence individuals' intentions to engage in domestic waste separation. Stoeva and Alriksson (2017) found that the degree to which individuals are satisfied with the actions of local authorities, even in areas not directly related to waste management, can significantly affect their decision to separate household waste. Furthermore, knowledge and the availability of information within the community are also important determinants. According to Welfens et al. (2016), the likelihood that individuals will engage in domestic waste separation increases when authorities provide education and accessible information about these processes. A recent review comparing multiple TPB-based studies on waste management, conducted by Hu et al. (2026), similarly suggests that education and information dissemination are associated with changes in pro-environmental behaviors within the context of the circular economy.

Examples of studies addressing waste separation using the TPB in environmental sciences across major countries are presented in Table 1.

**Table 1:** Examples of the use of TPB in environmental science and waste management.

Country	Reference	Issues
Thailand^*^	Apinhapath, 2014	Study based on the TPB, to understand recycling behavior and methods for changing people's habits
Iran^*^	Pakpour et al., 2014	Community waste management behaviors related to attitude, subjective norms, moral engagement, self-identity and intention.
Japan^**^	Hu et al., 2021	Use of questionnaires to determine the factors impacting citizens' intentions and actions toward waste segregation at the source.
China^*^	Zhang et al., 2015	Community waste segregation behaviors at the source: Using TPB in Guangzhou, China
Ethiopia^*^	Fikadu et al., 2022	Use of TPB to assess Butajira Town residents' intentions to adhere to SWM practices.
Ghana^*^	Boakye et al., 2024	Study of behavioral elements that impact waste bin acceptability and usage in Ghana.
Indonesia^*^	Abduh et al., 2018	Use of TPB to explain citizen waste separation behavior.
Spain^**^	Saldivia Gonzatti, 2021	Variables affecting the rate of collected sorted solid waste in Catalan municipalities.
Australia^**^	White and Hyde, 2012	The role that self-perceptions play in predicting Australian households' recycling habits.
Ecuador^*^	Hidalgo-Crespo and Amaya-Rivas, 2024	Pro-environmental actions of citizens in Guayas Province based on TPB.

^*^Developing countries. ^**^Developed countries. Own elaboration, adapted from Gallén and Peraita (2015).

More recently Novanda et al. (2026) propose that attitude, subjective norms, and perceived behavior control have a positive and significant influence on waste separation intention and the intention is a significant predictor of actual waste separation behavior. Hu et al. (2026) mentions that external factors like infrastructure and education support sustained behavioral change.

### 2.3 Theory of planned behavior in Latin America

The application of the TPB in Latin America has revealed that institutional and territorial factors are critical determinants that often override individual predispositions. For instance, Toledo et al. (2025), utilizing national household and municipal data from Ecuador, demonstrate that waste separation depends not only on demographic variables but also on key institutional factors such as the existence of differentiated collection systems, municipal regulations, and the implementation of collection fees. This study is fundamental to the present manuscript, as it validates those municipal and institutional variables, integrated here into our extended model, act as significant predictors of pro-environmental behavior within the regional context.

Furthermore, evidence from large metropolitan areas suggests that physical infrastructure conditions the effectiveness of subjective norms. Valenzuela-Levi and Flores (2023) observed in Santiago, Chile, that participation in recycling is mediated by both socio-territorial conditions and the proximity to disposal infrastructure (drop-off centers). Their findings reinforce the importance of our Perceived Behavioral Control variable, suggesting that a citizen's “perceived ease” is directly anchored to the technical and territorial feasibility provided by the municipality.

### 2.4 Public policy in Chile

In Chile, the geographical management of municipal solid waste (MSW) has historically been concentrated in urban areas, with a focus on waste characterization and the evaluation of sanitary landfills and informal dumping sites (Aliste Almuna, 2011). This pattern reflects broader state policies that tend to centralize decision-making in large urban centers, often driven by economic considerations (Montecinos, 2005). Similar dynamics can be observed in other policy domains, such as climate change, where centralization has contributed to delays in infrastructure development. For instance, the delayed implementation of an aqueduct project has been linked to centralized decision-making processes (Quinteros-Flores and Jorquera-Apablaza, 2019). In 1994, the National Environmental Commission (CONAMA) was established under Act No. 19,300, with the aim of coordinating environmental management at the national level. However, its scope did not specifically prioritize municipal solid waste (MSW) management, which at times limited progress in this area. In 1997, the concept of environmental sustainability was formally incorporated into Chilean legislation through the Environmental Impact Assessment System (Espinoza, 2001), as a result of coordinated efforts among multiple government ministries.

Subsequently, in 2006, the Integrated MSW Management Policy was introduced, establishing targets and monitoring mechanisms for waste management at the municipal level (CONAMA, 2005). Further progress was made with the enactment of Act No. 20,920—known as the Extended Producer Responsibility (EPR) Law—in 2016, which focuses on waste management and producer responsibility. From 2021 onwards, this law enabled the regulation of packaging and container design, introducing collection and recovery targets to be implemented starting in 2023 (Suazo Páez, 2017).

Recent studies suggest that these advances, aligned with circular economy principles, have contributed to improving perceptions of sustainability within the Chilean context (Valenzuela-Levi et al., 2024).

Despite legislative progress, such as the Extended Producer Responsibility Law (Ley REP), Chile faces significant structural challenges in municipal waste management. According to Valenzuela-Levi (2021), in a national study on municipal performance between 2013 and 2017, less than 0.8% of total domestic waste was selectively collected for recycling during that period. This study identifies that local recycling performance is strongly correlated with the population's educational level, service duration, the presence of composting programs, and regional disparities. Situating our research within this context allows for the interpretation of results not merely as individual choices, but as responses to the structural constraints and service gaps that characterize municipalities in southern Chile.

From a territorial and regional perspective, the Los Ríos Region in southern Chile presents a complex and challenging scenario regarding the management of household solid waste. Limited experience and capacity in environmental management have, in some cases, led municipalities to operate at or beyond legal limits in the management of disposal sites and landfills.

**Figure 2:**
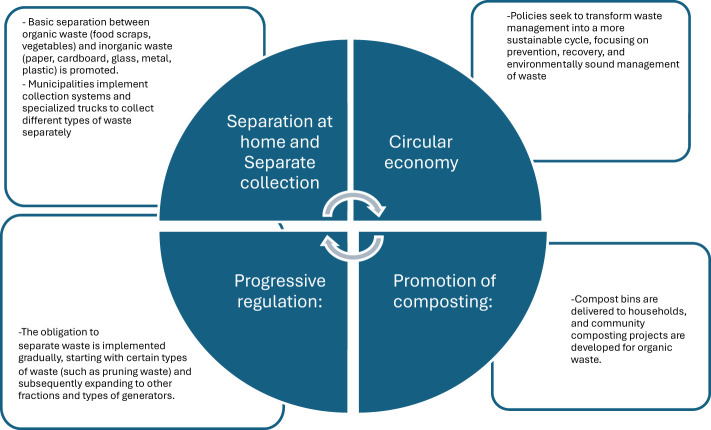
Key components of household waste management policies.

Public policies aimed at improving household waste management have primarily focused on promoting waste separation at the source—distinguishing between organic and inorganic materials—and on implementing specialized collection systems to support material recovery. As noted by De Kraker et al. (2019), these approaches are reflected in legislative and regulatory frameworks in countries such as Chile. Such measures include the development of differentiated collection systems, the distribution of composting infrastructure, and the gradual introduction of mandatory waste separation, particularly for organic waste and packaging (see Figure 2).

Information about waste types and related factors is essential for the effective implementation of the public policies presented in Figure 2. In this regard, Arkorful and Shuliang (2023) found that most participants in their study possessed accurate knowledge about waste, and that variables such as age and educational level were significant predictors of access to useful information supporting waste separation practices. As discussed in Figure 2, municipalities play a central role in implementing and improving waste collection systems. Within the decentralization framework proposed by Montecinos (2005), and consistent with the theory of multilevel governance developed by Hooghe and Marks (2001), strengthening local governance is closely associated with improved development outcomes.

In this context, administrative decentralization enables municipalities to assume greater responsibility for managing social services, including waste collection and separation. A decentralized system may also enhance accountability to citizens in relation to waste disposal and treatment. For waste separation systems to function effectively, regional governments in Chile require greater budgetary flexibility to invest in collection infrastructure and waste management facilities.

More broadly, waste management in Chilean households may benefit from governance arrangements that allocate decision-making authority and financial resources to regional and local levels, allowing waste separation solutions to be designed and implemented in ways that are responsive to local conditions, rather than relying exclusively on centralized, sector-based approaches.

## 3 Research hypothesis

**Figure 3:**
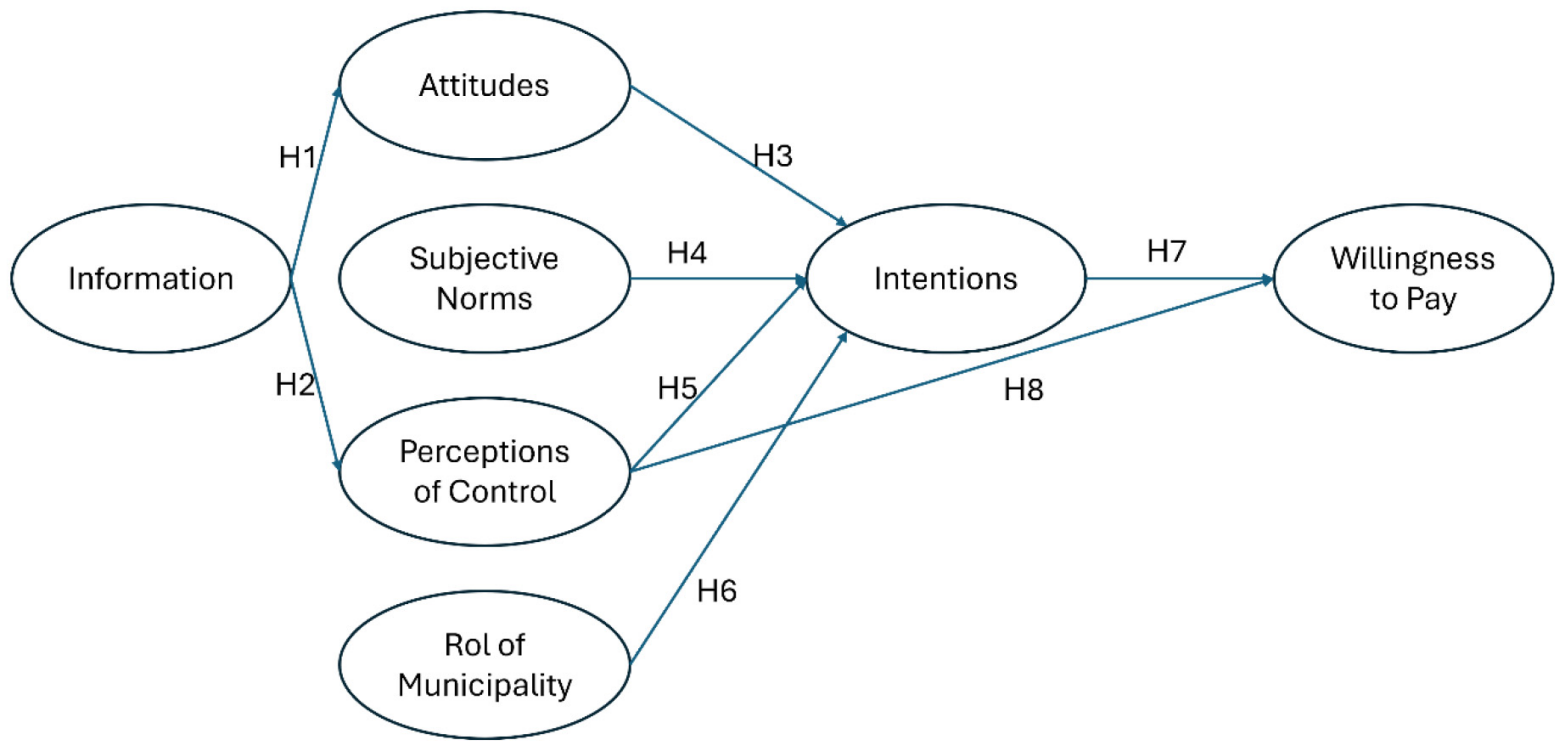
Research model.

Figure 3 shows our research model, which is based on the following hypotheses. The specific hypotheses regarding waste separation are stated below.

H1. Information is positively associated with attitudes toward waste separation at source.

H2. Information is positively associated with perceived behavioral control toward waste separation at source.

H3. Attitudes are positively associated with the intention to separate waste at source.

H4. Subjective norms are positively associated with the intention to separate waste at source.

H5. Perceived behavioral control is positively associated with the intention to separate waste at source.

H6. The perceived role of the municipality is positively associated with the intention to separate waste at source.

H7. The intention to classify domestic waste is positively associated with willingness to pay for a service that recycles or recovers previously separated waste.

H8. Perceived behavioral control is positively associated with willingness to pay for a service that recycles or recovers separated waste.

## 4 Methods, sample and measurements

This study was conducted in three cities in southern Chile, Coyhaique, Panguipulli, and Valdivia, where formal municipal waste separation systems are either limited or absent. In these contexts, most household waste is disposed of in landfills, making them relevant settings for analyzing behavioral intentions toward waste separation. Data collection took place between March and September 2024.

A non-probabilistic convenience sampling strategy was employed. Participants were recruited *in situ* in the selected cities (Coyhaique, Panguipulli, and Valdivia) during the data collection period. Eligibility criteria required participants to be at least 18 years old and residents of the selected cities. A total of 302 valid responses were obtained after excluding incomplete or inconsistent questionnaires. Due to the nature of in-person recruitment, a response rate could not be calculated.

The study was conducted in accordance with the Declaration of Helsinki. Informed consent was obtained from all 302 participants prior to their participation via a digital questionnaire (Microsoft Forms). Data was processed anonymously to ensure participant confidentiality. The final sample exhibited a gender imbalance, with 88.1% of respondents identifying as female. This may reflect the gendered nature of household waste management practices, as suggested in previous research. A t-test was performed to check for gender differences across main variables, its results are discussed in the next section.

The adequacy of the sample size (*n* = 302) was evaluated based on established guidelines for confirmatory factor analysis (CFA) and structural equation modeling (SEM). The sample exceeds the commonly recommended minimum threshold of 200 observations for SEM, which is considered sufficient for stable parameter estimation and reliable model fit assessment (Kline, 2016; Hair et al., 2019).

Additionally, the sample size satisfies the recommended ratio of observations to estimated parameters, which typically ranges from 10:1 to 15:1. Given the number of latent constructs and observed indicators included in the model, the available sample ensures adequate statistical power to detect medium effect sizes at conventional significance levels (Cohen, 1988).

Overall, the sample size is deemed sufficient to support both the measurement model (CFA) and the structural model (SEM) analyses conducted in this study.

A measurement instrument (questionnaire) was designed following the guidelines proposed by Icek Ajzen (Ajzen, 2006, 2019). The questionnaire was administered in Spanish, as all participants were native Spanish speakers. The original items were adapted from previously validated scales and carefully reviewed by the research team to ensure linguistic clarity and contextual relevance for the Chilean setting. Rather than applying a formal back-translation procedure, the instrument underwent a pilot test with 20 participants from the sampled cities, which allowed the identification and correction of minor wording issues and ensured that all items were clearly understood by respondents. The instrument consisted of 31 closed-ended questions measured on a 5-point Likert scale (1 = “strongly disagree” to 5 = “strongly agree”) and was organized into eight sections: (A) demographic profile; (B) attitudes toward waste separation at source; (C) subjective norms; (D) perceived behavioral control; (E) intention to separate waste at home; (F) willingness to pay; (G) role of government and municipality; and (H) information.

The items used in this study were adapted from previously validated and widely used scales, as presented in Table 2. A list of the items used in this study can be found in Table 4.

**Table 2:** Model variables and references.

Variables of the model	References
Attitudes	Ma et al., 2018; Shen et al., 2019; White and Hyde, 2012; Zhang et al., 2015
Subjective norms	Ma et al., 2018; Shen et al., 2019; White and Hyde, 2012; Zhang et al., 2015
Perceptions of control	Ma et al., 2018; Shen et al., 2019; White and Hyde, 2012; Zhang et al., 2015
Role of the municipality	Mohanty et al., 2021
Intentions	Ma et al., 2018; Shen et al., 2019; White and Hyde, 2012; Zhang et al., 2015
Willingness to pay	Anebagilu et al., 2021; Kumar and Nandini, 2013; Vassanadumrongdee and Kittipongvises, 2018
Information	Anebagilu et al., 2021; Kumar and Nandini, 2013; Vassanadumrongdee and Kittipongvises, 2018; Zhang et al., 2015

**Table 4:** Items, scale reliability and CFA factor loadings.

Scales	Factor Loading
Attitudes toward waste separation at source (α = 0.84)	
I believe that separating waste at home for recycling is important for environmental protection	0.81
I would like to separate waste at home in order to recycle it	0.90
Separating waste at home would help preserve natural resources	0.65
Separating waste would reduce the amount of waste sent to landfills	0.58
Participating in waste separation activities improves the quality of life in the community	0.53
Subjective norms (α = 0.78)	
I believe that my family and friends would support me in separating waste	0.78
I believe that my neighbors would support me in separating my waste	0.82
People who are important to me think that I should separate waste at home	0.67
*Perceived behavioral control (α = 0.72)*	
I believe it would be easy for me to separate waste at home	0.68
I believe that lack of time is a barrier to separating waste at home	0.79
I believe that I have the necessary knowledge to separate waste correctly at home	0.73
Intention to separate waste at home (α = 0.75)	
I intend to separate waste at home	0.78
I will make an effort to separate waste at home in the future	0.84
I plan to separate waste at home on a regular basis	0.64
Willingness to pay (α = 0.80)	
I am willing to pay a monthly fee for the municipality to process the waste I separate at home	0.61
I am willing to pay for waste separation services if it improves waste management in my municipality	0.92
Paying for waste separation is an investment in the future of the environment	0.77
Role of government and municipality (α = 0.82)	
I believe that the municipality would do a good job processing the waste separated by residents	0.63
I believe that the current municipal infrastructure encourages me to separate waste	0.71
I am satisfied with the actions taken by the municipality regarding waste separation programs	0.69
Information (α = 0.77)	
The municipality provides me with accurate information about the types of materials I can recycle	0.86
The municipality provides sufficient information about the available infrastructure for disposing of separated waste	0.83
The information provided by the municipality about waste separation is clear and easy to understand	0.89

## 5 Results and discussion

### 5.1 Evaluation of measurements (CFA)

A total of 302 respondents completed the survey, and the demographic characteristics of the final sample are presented in Table 3. Confirmatory Factor Analysis (CFA) was employed to assess the measurement model as part of the two-stage Structural Equation Modeling (SEM) approach. CFA enabled evaluation of the fit between the theoretical model and the observed data (Tharenou et al., 2012), examining the factor loadings of each item within their respective constructs to ensure that items accurately reflected the underlying theoretical concepts. The CFA included seven latent constructs and 23 observed variables, analyzed using AMOS v29.0. The overall model demonstrated satisfactory fit, with χ^2^/ df = 3.85, CFI = 0.91, IFI = 0.90, GFI = 0.90, NFI = 0.93, standardized RMR = 0.076, and RMSEA = 0.055, all within acceptable ranges (Green, 2016; Hair et al., 2019).

**Table 3:** Respondents' demographics.

Gender	Number	Percentage from total
Male	36	11.9
Female	266	88.1
Other	0	0.0
Age		
34 and less	51	16.9
Between 35 and 44	66	21.9
Between 45 and 54	136	45.0
More than 55	49	16.2
Level of education		
No formal studies	10	3.3
Primary school	43	14.2
High school	133	44.0
University	116	38.4
City		
Coyhaique	96	31.8
Panguipulli	19	6.3
Valdivia	187	61.9
Household		
House	286	94.7
Flat	6	2.0
Shared house	10	3.3

Convergent validity was confirmed, as all factor loadings exceeded 0.5 (Hair et al., 2019; Tharenou et al., 2012). Scale reliability, assessed via Cronbach's alpha, also exceeded the recommended threshold of 0.7 (Hair et al., 2019), indicating high internal consistency among items within each construct. Items, scale reliability and factor loadings are presented in Table 4. Discriminant validity was evaluated using the average variance extracted (AVE), with the square roots of AVE (shown on the diagonal in Table 5) exceeding both 0.5 and the inter-construct correlations, confirming discriminant validity (Hair et al., 2019; Tabachnick and Fidell, 2019). Composite reliability (CR) values are also reported and exceeded the 0.7 threshold recommended by Hair et al. (2019).

**Table 5:** Discriminant validity assessment.

Variable	CR	AVE	Attitudes	Subjective norms	Perceptions of control	Willingness to pay	Information	Role of municipality	Intentions to separate waste
Attitudes	0.827	0.500	**0.707**						
Subjective norms	0.864	0.777	0.334^**^	**0.881**					
Perceptions of control	0.723	0.503	0.190^**^	0.298^**^	**0.709**				
Willingness to pay	0.815	0.602	0.085	−0.105	0.062	**0.776**			
Information	0.752	0.570	0.204^**^	0.212^**^	0.092	0.083	**0.755**		
Role of municipality	0.705	0.505	0.368^**^	0.425^**^	0.208^**^	−0.043	0.303^**^	**0.710**	
Intentions to separate waste	0.703	0.546	0.290^**^	0.469^**^	0.336^**^	−0.050	0.177^**^	0.337^**^	**0.739**

AVE: Average Variance Extracted and Correlation Estimates. The non-diagonal elements show the latent correlations, whereas the bold values in the diagonal represent the square root of the AVE. CR: Composite Reliability. ^*^ indicates statistical significance at the *p* < 0.05 level. ^**^ shows significance at *p* < 0.01 level.

To assess potential gender bias in the sample, independent samples *t*-tests were conducted comparing male and female respondents across the main study variables. Given that Levene's test indicated unequal variances in some cases, results were interpreted using the “equal variances not assumed” estimates. The results showed no statistically significant differences between male and female respondents in attitudes (*p* = 0.725), subjective norms (*p* = 0.948), perceived behavioral control (*p* = 0.816), willingness to pay (*p* = 0.151), information (*p* = 0.438), or perceptions of the municipality's role (*p* = 0.170) (Table 6). These findings suggest that, despite the gender imbalance in the sample, the main constructs of the model do not significantly differ across gender groups, supporting the robustness of the results.

**Table 6:** Gender differences across main variable.

Variable	Male (*n* = 36) mean	Female (*n* = 266) mean	*t*-value	*p*-value
Attitudes	4.32	4.38	−0.35	0.725
Subjective Norms	3.63	3.61	0.07	0.948
Control	3.29	3.26	0.23	0.816
Willingness to Pay	3.24	3.53	−1.46	0.151
Information	4.07	4.17	−0.78	0.438
Municipality Role	3.96	4.14	−1.39	0.17

### 5.2 Results of hypotheses testing

A structural model was built to test hypotheses after CFA was satisfactorily completed. Normality for each item was tested through plots, and skewness and kurtosis were within acceptable ranges (±2) (Gravetter and Wallnau, 2014; Hair et al., 2019). Histograms also confirmed normality. The model was controlled by respondents' hometown, household type, level of education, age and gender, all highly relevant in rural contexts and consistent with other studies in this field (e.g., Vassanadumrongdee and Kittipongvises, 2018; Zhang et al., 2021).

**Figure 4:**
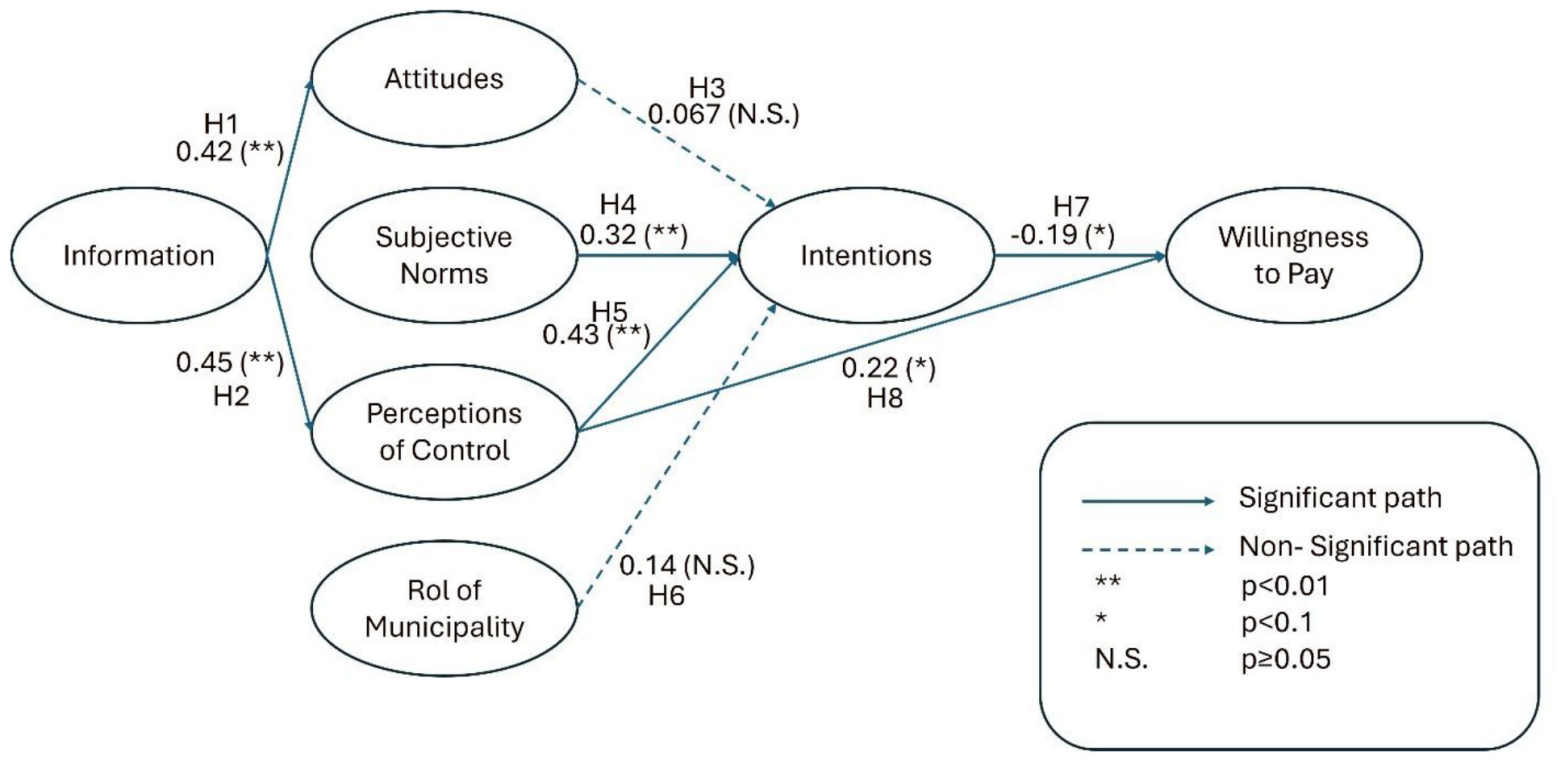
Results and significance for each hypothesis.

Table 7 depicts correlations between model and control variables. The goodness of fit of the structural model was satisfactory, with values for χ2/ df = 3.80, CFI = 0.92, IFI = 0.92, GFI = 0.90, NFI = 0.90, Standardized RMR = 0.088 and RMSEA = 0.067, all of them within acceptable thresholds (Green, 2016; Hair et al., 2019). Figure 4 shows the model and the results obtained.

**Table 7:** Correlations.

Type of variable	Variable number	Name of variable	Mean	SD	1	2	3	4	5	6	7	8	9
Model variables	1	Attitudes	4.370	0.599									
	2	Subjective norms	3.616	0.904	0.334^**^								
	3	Perceptions of control	3.260	0.818	0.190^**^	0.298^**^							
	4	Willingness to pay	3.499	0.901	0.085	−0.105	0.062						
	5	Information	4.157	0.646	0.204^**^	0.212^**^	0.092	0.083					
	6	Role of municipality	4.119	0.583	0.368^**^	0.425^**^	0.208^**^	−0.043	0.303^**^				
	7	Intentions to separate waste	3.881	0.790	0.290^**^	0.469^**^	0.336^**^	−0.050	0.177^**^	0.337^**^			
Control variables	8	Sex	0.881	0.325	0.030	−0.004	−0.014	0.106	0.050	0.099	−0.017		
	9	Age	3.606	0.951	0.064	−0.146^*^	−0.054	0.040	−0.020	−0.023	−0.058	0.116^*^	
	10	Respondents' levels of education	3.175	0.794	0.140^*^	0.349^**^	0.096	−0.132^*^	0.037	0.208^**^	0.283^**^	−0.215^**^	−0.295^**^

^*^p < 0.05; ^**^p < 0.01. Student's gender: 0 for males; 1 for females. Respondents' levels of education: 1–5 scale, from primary education to postgraduate degree.

The model explains a substantial proportion of variance in behavioral intention (*R*^2^ = 0.357), indicating that attitudes, subjective norms, perceived behavioral control, and perceived municipal role jointly account for 35.7% of the variance in intention.

The model also explains variance in perceived behavioral control (*R*^2^ = 0.249) and attitudes (*R*^2^ = 0.262), reflecting the influence of information and socio-demographic variables. Lower explanatory power was observed for willingness to pay (*R*^2^ = 0.046), suggesting that additional factors may influence this outcome.

The results found for H1 were significant (β = 0.42, *p* < 0.01): there was a strong association between levels of information about household waste separation provided by municipalities and attitudes toward waste separation.

Results for H2 also showed a significant relationship (β = 0.45, *p* < 0.01) between levels of information about household waste separation provided by municipalities and perceptions of control toward waste separation.

H3 was not supported, since the effect of attitudes toward waste separation on intentions of classifying household waste was not significant (β = 0.067, *p* >0.05).

Findings for H4 and H5 suggested that from the elements proposed by the TPB, subjective norms (β = 0.32, *p* < 0.01) and perceptions of control (β = 0.43, *p* < 0.01) are the most important predictors of the intention of classifying household wastes.

Results for H6 show that it is not supported, since the effect of the perceived role of municipality on household waste separation on the intentions of classifying waste is not statistically significant (β = 0.14, *p* >0.05).

Interestingly, H7 is not supported, since the relationship between intentions to classify household waste and the willingness to pay for waste collection is significant but negative (β = −0.19, *p* < 0.05). Finally, H8 is supported since the relationship between perceptions of control and willingness to pay appears to be positive and significant (β = 0.22, *p* < 0.05).

### 5.3 Indirect effects

Indirect effects were examined to assess the mediating role of the TPB constructs. To formally test mediation effects, bootstrap procedures with 5,000 resamples and bias-corrected 95% confidence intervals were estimated within the SEM framework. The results indicate that information has a substantial indirect effect on behavioral intention [β = 0.332, 95% CI (0.12, 0.41)], primarily mediated through perceived behavioral control. Specifically, information significantly predicts perceived behavioral control, which in turn exerts a strong positive association with intention. The bootstrap confidence intervals for this indirect pathway did not include zero, supporting the statistical significance of the mediation effect.

In contrast, although information significantly influences attitudes, attitudes do not significantly predict intention, suggesting that the mediating role of attitudes is not supported in this context. Additionally, information exhibits a smaller indirect effect on willingness to pay (β = 0.092), reflecting the combined influence of perceived control (positive effect) and behavioral intention (negative effect). These findings highlight the complex and indirect pathways through which information shapes both pro-environmental intentions and economic preferences.

### 5.4 Common method bias

Given that all variables were collected through a self-reported questionnaire in a single survey wave, common method bias was assessed using Harman's single-factor test. The results indicate that the first factor accounts for 19.72% of the total variance, which is well below the recommended threshold of 50%. This suggests that common method bias is unlikely to pose a serious threat to the validity of the findings. In addition, multiple factors emerged with eigenvalues greater than one, further supporting the multidimensionality of the constructs and reducing concerns about common method variance.

### 5.5 Multicollinearity

Multicollinearity was assessed by examining variance inflation factors (VIF) and tolerance values for the predictors of behavioral intention. The results show that VIF values ranged from 1.11 to 1.34, well below the recommended threshold of 5, while tolerance values ranged from 0.75 to 0.90, exceeding the minimum acceptable level of 0.20. These results indicate that multicollinearity is not a concern in this study.

## 6 Discussion

The results of the structural model provide nuanced insights into the factors associated with waste separation intentions within a municipal context, offering both confirmation and extension of the Theory of Planned Behavior. Consistent with the core assumptions of TPB, perceived behavioral control (β = 0.43, *p* < 0.01) and subjective norms (β = 0.32, *p* < 0.01) are strongly associated with waste separation intentions. These findings suggest that intentions to recycle are closely linked to perceptions of feasibility and social embeddedness, rather than solely to evaluative judgments. In practical terms, individuals report stronger intentions to recycle when they perceive access to resources, infrastructure, and capabilities, and when they perceive support or expectations from their immediate social environment.

In contrast, attitudes toward waste separation are not significantly associated with intentions. While this finding appears to diverge from the standard assumptions of the Theory of Planned Behavior, it is consistent with a growing body of research reporting context-dependent effects of attitudes in pro-environmental behavior. One possible explanation lies in the distribution of the attitudinal construct in the present sample. Descriptive results indicate relatively high mean values for attitudes (above 4 on a 5-point scale), indicating generally favorable evaluations of waste separation behavior, along with the presence of limited variance or potential ceiling effects, which may reduce their explanatory power in the structural model.

Additionally, this pattern has been observed in prior studies where attitudes fail to significantly predict waste separation intentions when structural or contextual barriers are salient (Knussen et al., 2004; Barr and Gilg, 2007; Wan et al., 2014). In such cases, perceived behavioral control and situational constraints tend to play a more central role in shaping intention. This interpretation is particularly relevant in the context of the cities analyzed in this study, where infrastructure and service provision for waste separation remain uneven. Taken together, these findings suggest that the relationship between attitudes and intention may be contingent on both measurement characteristics (e.g., variance) and contextual conditions, rather than representing a uniform or universal effect. The role of information emerges as an important indirect factor within the model. Municipal information provision is positively associated with both attitudes (β = 0.42, *p* < 0.01) and perceived behavioral control (β = 0.45, *p* < 0.01), indicating that information is linked both to how individuals evaluate waste separation and to how capable they feel of performing it. These findings highlight the relevance of communication strategies that not only raise awareness but also reduce uncertainty regarding how, where, and when to recycle. In this sense, information appears to be associated with both cognitive and practical dimensions of intention formation.

A noteworthy result is the non-significant relationship between the perceived role of the municipality and waste separation intentions. This suggests a potential decoupling between institutional perceptions and individual decision-making. Rather than being directly associated with intention, the role of the municipality may be perceived as a background condition or a taken-for-granted responsibility. This pattern may indicate that institutional presence alone is insufficient to be associated with stronger intentions in the absence of perceived improvements in infrastructure or direct engagement with households.

The analysis of willingness to pay (WTP) reveals an interesting pattern. Waste separation intentions are negatively associated with willingness to pay (β = −0.19, *p* < 0.05), indicating that individuals reporting stronger waste separation intentions also report lower willingness to financially support related services. One possible interpretation is that individuals with stronger pro-environmental orientations may view waste management as a public good that should be financed collectively rather than through direct user fees. At the same time, perceived behavioral control is positively associated with WTP (β = 0.22, *p* < 0.05), suggesting that individuals who perceive greater capability to engage in waste separation may also be more open to contributing financially, possibly because they anticipate making use of such services.

Taken together, these findings extend TPB in several ways. First, they suggest that the association between attitudes and intentions may be context-dependent, particularly in environments characterized by structural constraints. Second, they highlight the central role of perceived behavioral control as a key factor associated with both intentions and willingness to pay. Third, they incorporate willingness to pay as a complementary outcome, revealing potential tensions between pro-environmental intentions and economic preferences that are not typically addressed in standard TPB applications.

From a policy perspective, these findings should be interpreted with caution. Given the cross-sectional design of the study, the results identify associations rather than causal relationships or observed behavioral change. Nevertheless, the patterns observed suggest that factors such as infrastructure availability, social influence, and access to information are closely linked to waste separation intentions. Interventions aimed at reducing structural barriers, strengthening community norms, and improving the clarity and accessibility of information may therefore be relevant areas for policy consideration. In addition, the observed divergence between behavioral intention and willingness to pay suggests that financing mechanisms should be carefully designed, as strong pro-environmental intentions do not necessarily correspond to a greater willingness to bear direct costs.

## 7 Conclusion

This study applied the Theory of Planned Behavior to examine the factors associated with waste separation intentions among residents in cities in southern Chile, with the broader aim of understanding how individuals relate to an emerging and increasingly institutionalized waste management system. The findings indicate that subjective norms and perceived behavioral control are more strongly associated with intention than attitudes, highlighting the relevance of social dynamics and perceived feasibility in this context.

A key contribution of this study lies in identifying the role of municipal information as an important indirect factor within the model. Information provision is positively associated with both attitudes and perceived behavioral control, suggesting that access to clear and relevant information is linked to how individuals evaluate waste separation and to how capable they feel of engaging in it. This finding is particularly relevant in the context of ongoing policy developments such as the Extended Producer Responsibility (EPR/REP) framework, where citizen participation is a central component. At the same time, the results reveal a divergence between waste separation intentions and willingness to pay, indicating that stronger pro-environmental intentions may coexist with lower willingness to financially support related services.

Several additional contributions are worth highlighting. First, although many studies refer to the “municipal” level (e.g., Wang et al., 2021), they often do not incorporate actual municipal performance or citizens' perceptions of the municipality as measurable variables. This study addresses that gap by explicitly including these dimensions in the empirical model.

Second, the study directly links the determinants of individual behavior to municipal public policies. In particular, it examines how perceived institutional support, operationalized through the clarity of information and the adequacy of infrastructure provided by local authorities, acts as a relevant antecedent of behavioral intention.

Third, while the Theory of Planned Behavior traditionally focuses on three core constructs, this study extends the framework to better capture the socio-institutional context. Specifically, it incorporates information availability, the perceived role of municipalities, and willingness to pay (WTP) into the structural equation model.

Fourth, in contrast to prior studies such as (Ma et al. 2018), the findings indicate that attitudes play a limited or non-significant role in predicting intentions in the Chilean context. This suggests that in environments characterized by structural constraints, subjective norms and perceived behavioral control may be more influential than individuals' evaluative judgments.

Fifth, whereas much of the existing literature focuses on developed countries with well-established waste separation systems or on large metropolitan areas in developing contexts, this study examines medium-sized cities in southern Chile (specifically Coyhaique, Panguipulli, and Valdivia) where formal municipal waste separation systems are still under development.

Beyond its empirical results, this study illustrates the usefulness of TPB as a framework for analyzing behavioral intentions in environmental contexts. The model allows for the identification of key psychological and contextual factors associated with intention, which may inform the design and evaluation of policy interventions. In this sense, TPB-based indicators could serve as complementary tools for monitoring the implementation of waste management strategies at the local level.

However, several limitations should be acknowledged. First, the cross-sectional design of the study does not allow for causal inference or the observation of behavioral change over time; therefore, the findings should be interpreted as associations among latent constructs and stated intentions. Second, the use of self-reported data may introduce common method bias, although statistical checks suggest that this is unlikely to be a major concern. Third, the model does not fully capture broader structural and contextual influences, such as socioeconomic conditions, institutional trust, or the distribution of responsibilities among stakeholders.

Future research could extend this work by incorporating additional variables, including sociodemographic and contextual factors, and by testing alternative specifications of TPB. In particular, distinguishing between different institutional actors—such as national government and municipalities—may provide a more nuanced understanding of how responsibility and accountability are perceived. Furthermore, integrating constructs related to civic responsibility or environmental citizenship may enhance the explanatory scope of the model. Longitudinal or experimental designs would also be valuable to better assess causal mechanisms and behavioral change over time.

In summary, this study contributes to the literature by showing that waste separation intentions are more closely associated with perceived feasibility, social influence, and access to information than with attitudes alone. While these findings do not establish causal relationships, they provide useful insights into the factors linked to pro-environmental intentions in the Chilean context and may serve as a basis for future research and policy discussion.

## Data Availability

The raw data supporting the conclusions of this article will be made available by the authors, without undue reservation, upon reasonable request starting 3 years after the publication date of this article. Requests should be directed to Jean Pierre Doussoulin (jdoussoulin@gmail.com).
